# Course Review: Doctors Academy Basic Sciences and Clinical Application for the Membership of the Royal College of Surgeons Part A Course

**DOI:** 10.7759/cureus.19804

**Published:** 2021-11-22

**Authors:** Khemerin Eng, Poh Hong Tan

**Affiliations:** 1 General Surgery, St. James's University Hospital, Leeds, GBR; 2 Plastic Surgery, Wythenshawe Hospital, Manchester, GBR

**Keywords:** royal college of surgeons, professional exam, continuing health education, mrcs, educational course

## Abstract

The Membership of the Royal College of Surgeons (MRCS part A and B) is a mandatory examination that is required by all trainees to enter the surgical speciality training programme in the United Kingdom. Students and clinicians alike often find it a struggle to succeed in passing these exams given the breadth and depth of knowledge required across a spectrum of surgical sciences. There are several factors contributing to success, including ethnic background, number of attempts, and medical school performance. Studies have also shown that a marker of success for Part B was influenced by the Part A performance. During the COVID-19 peak, the MRCS Part A written exam has had its format changed to an online exam reflecting the policy surrounding social distancing. Due to this, the pass mark has become more difficult to attain compared to traditional face-to-face settings. As such, many trainees are finding it more difficult to succeed in the exam, leading to more emphasis on more thorough preparation for exams. This can be in the form of courses, textbooks, and using more of the online question banks.

The Basic Sciences and Clinical Application for MRCS A Course is organised by the Doctors Academy group and details an intensive and interactive three-day revision course held intermittently throughout the year in Cardiff, Wales multiple times a year. Delivered by an expert faculty, it aims to provide maximum preparation in the lead up to the MRCS Part A exam with emphasis on retaining high-yield knowledge and application of key exam revision techniques. We reviewed the post-course surveys from 2019 to 2021 and found that the majority of attendees found the course content to be excellent and relevant to the exams. These questions were a mandatory part of the course and reflect a variety of training grades from foundation to core training/speciality training (CT1/2 to ST1/2). Overall, candidates found that this course aided their exam preparation immensely and contributed to passing.

## Introduction and background

The Membership of the Royal College of Surgeons (MRCS) of Great Britain and Ireland is a postgraduate diploma awarded to those that pass the intercollegiate membership examination. It is mandatory for all surgical trainees to membership into one of the four royal colleges in the United Kingdom and Ireland and comprises one of the essential entry barriers into higher speciality training [1]. The exam has two parts, a written paper (part A) and an objective structured clinical examination (part B).

Part A is a five-hour multiple-choice question with a pass rate of approximately 35-37 per cent [2], for many applicants, this exam acts as a barrier to surgical training as it tests basic surgical sciences and applied clinical anatomy at much greater depth compared to most United Kingdom medical school finals. There has been evidence that suggests the follow-up exam, or Part B, has independent factors contributing to success, which includes passing the Part A exam with fewer attempts [3]. This highlights the importance of applicants preparing well in advance for the broad content areas as well as being able to transition the knowledge towards the 18 stations Part B [4].

## Review

Who should attend?

Delegates attending the 2019-2021 courses came from varied surgical backgrounds, ranging from foundation doctors (junior house officers) to senior house officers, including ST1/2 (registrar) trainees in neurosurgery, orthopaedics, ENT and general surgery. The Doctor’s Academy MRCS A course is a three-day course hosted in the College of Physicians and Surgeons of Cardiff, Cardiff, Wales. Close to the train station, the venue is easy to navigate via public transport. Centrally placed, it allows for various local accommodations surrounded by city atmosphere, including shopping malls, restaurants and bars. Each event averaged 34 delegates with limited positions available at a cost of £375, with lunch and coffee breaks provided each day. A total of 100% of delegates regarded the venue, facilities as well as refreshments to be excellent (48%) and very good (52%). There are various factors involved that can influence success at these entrance exams, including ethnicity, grade or training level, past medical school performance and indeed a number of attempts at Part A [5]. Those wishing to maximise their revision opportunities may find this course to be beneficial.

Aims

The aim of the Basic Sciences and Clinical Application of MRCS A is to develop a strong foundation and understanding of core components of the MRCS part A exam including surgical anatomy, system-specific pathology, and applied surgical sciences. This includes regular peer-to-peer discussion, exam-style question sessions and emphasis on MRCS A sitting technique. The course is designed by Professor Stuart Enoch, specialist plastic surgeon and expert medical educator, and provides a combination of didactic lectures, curated MRCS A style multiple-choice questions, and interwoven topic discussion to enhance recall. Faculty tutors included numerous consultant and PhD fellows across a wide range of surgical specialities, including orthopaedics, vascular, neuroanatomy, and anaesthetics.

 

Course content

**Figure 1:**
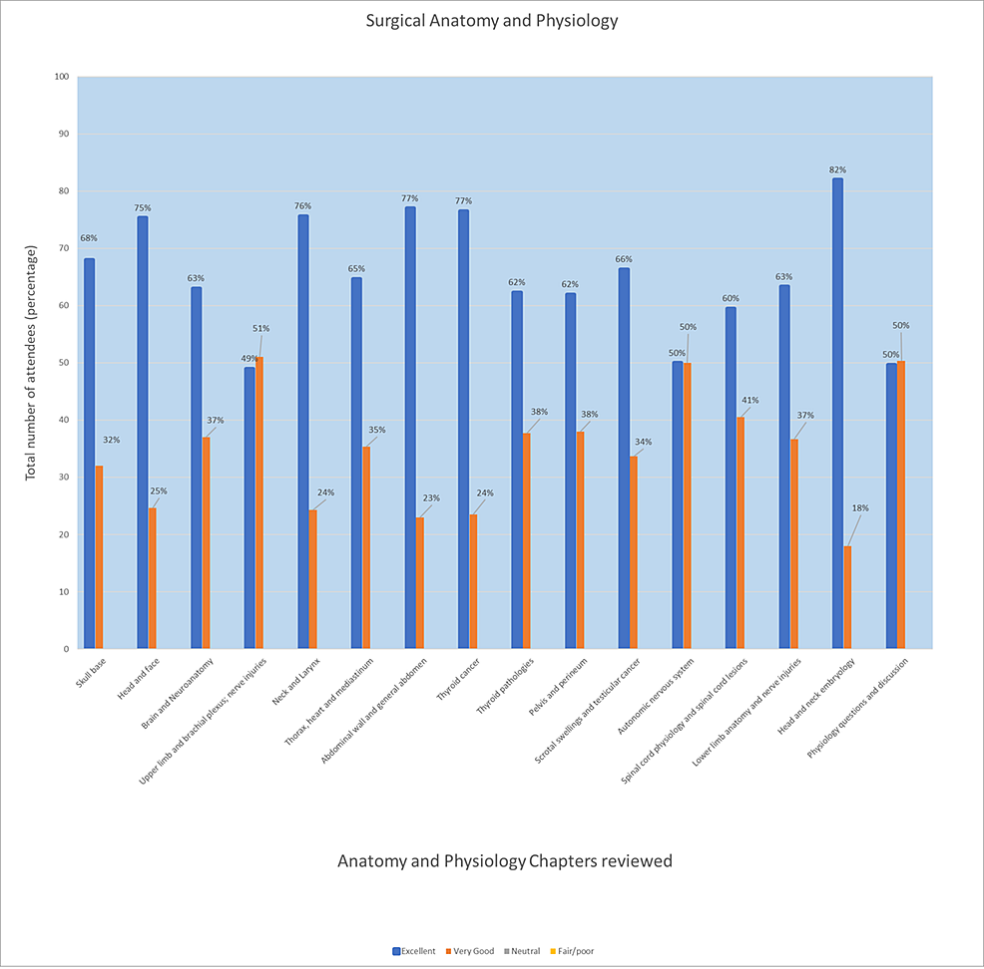
Feedback of attendees for the surgical anatomy and physiology topics covered (2019-2021). Anatomy is often the favoured topic of the exam, with clinicians likely to do well here compared to the applied surgical sciences. The majority of all attendees found the bulk content of the course to be excellent, with no negative feedback regarding these particular chapters. Overall, the four feedback options ranged from fair/poor to neutral, very good, and excellent.

Day 1 began with an introduction from Professor Enoch, who outlined the various topics covered each day. Delegates were provided with a zipper case folder with complimentary stationery and appropriate resources for note taking and highlighting. With an emphasis on surgical anatomy and pathology, day 1 comprised a systematic approach to high-yield scenarios and topics including top-to-toe organ, musculoskeletal and neurovascular components. A total of 99% of the delegates found the surgical anatomy and physiology content to be relevant to the exam, excellent (69%) or very good (31%) (Figure 1) and appreciated the opportunities to discuss topics and sample questions. The faculty provide scheduled breaks allowing for refreshments and opportunities for attendees to have one-to-one question time with staff regarding the exam.

**Figure 2:**
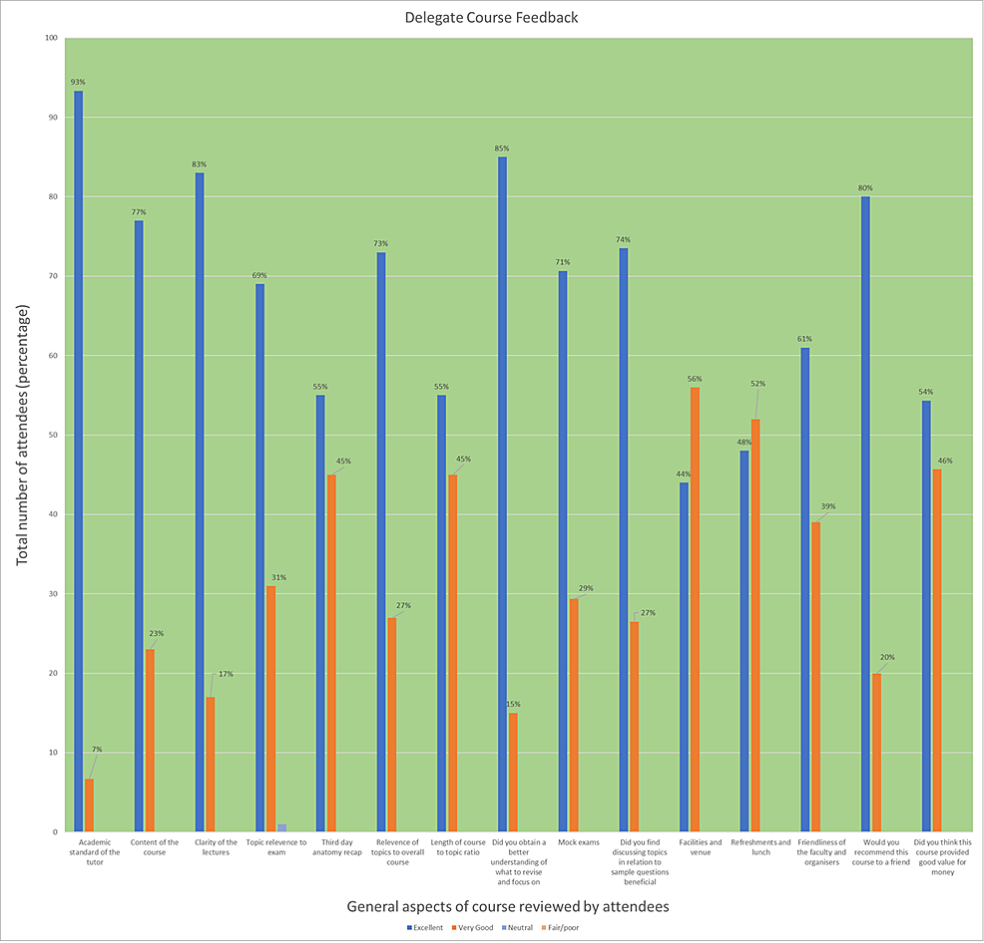
Feedback from attendees regarding the overall course from 2019-2021. Doctor's Academy carried out post-course surveys for each attendee in order to gain feedback and review the results. They have been graphed here and an overwhelming majority of attendees had excellent opinions on the structure and management of the course. This included stringent feedback on topics such as catering, access to the tutors, friendliness of faculty and whether attendees felt the course content was relevant or not. When reviewing the course, attendants were able to rank from fair/poor, to neutral, very good and excellent.

Day 2 involved a shift of focus towards surgical pathology and a recap of the previous day’s contents, including mock exams. One of the highlights involved splitting into groups and engaging in semi-competitive trivia-style activities in between lectures. Topics included embryology, multi-system cancer pathology, and microbiology. Conceptual videos, diagrams and interactive question-and-answer sessions were employed to enhance absorption of the topics. The second half of the day focussed on essential statistical topics in the exam as well as tips and techniques for sitting the five-hour exam. Every attending delegate appreciated the clarity of lectures (excellent 83% or very good 27%) and considered the content to be highly useful for revision (excellent 85% or very good 15%) (Figure 2).

**Figure 3:**
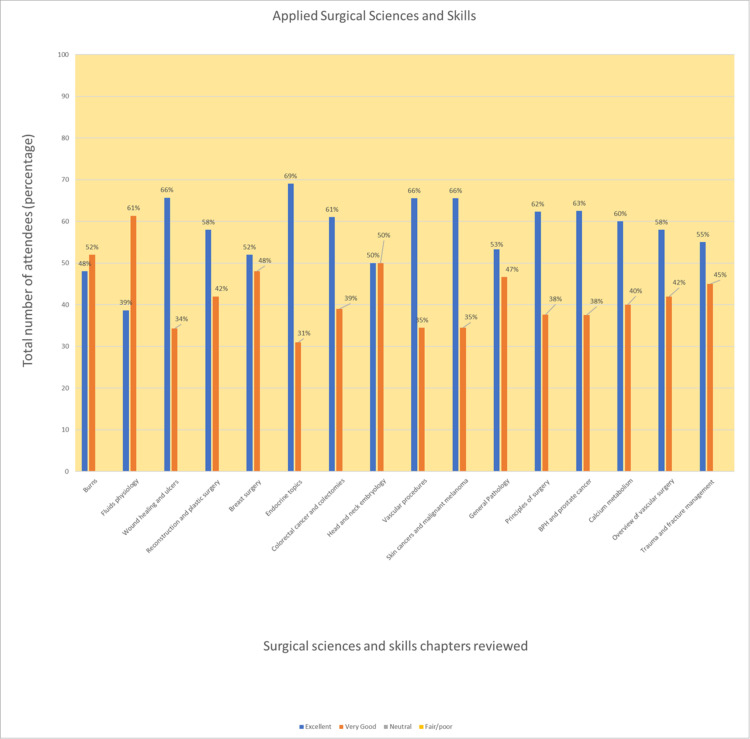
Feedback of attendees regarding the surgical sciences and skills section (2019-2021). Making up more than half the Part A exam content, the applied surgical sciences aspect remains a challenging topic for most examinees. This resulted in more equal feedback from the attendees with a more equal weighting between "excellent" and "very good" for the topics covered. The other two possible options included fair/poor and neutral, of which there were no votes for these in the surveys.

The whole of Day 3, the final day, was dedicated to principles of surgery and applied sciences. This included advanced trauma life support (ATLS) principles, pre-operative and post-operative management, surgical fundamentals surrounding sutures, wound healing, and post-op complications. There was a greater emphasis on revising the course’s curriculum and consolidating knowledge learnt over the three days. All delegates rated the third-day anatomy recap as well as relevant to the overall course as excellent/very good (Figure 3). Staff faculty ended the course with an opportunity for course feedback as well as providing a certificate to each attendant.

## Conclusions

Overall, it was generally agreed upon by all the delegates that this course was excellently run, delivering relevant core conceptual knowledge-driven home with an intensive mock exam revision setting. The exhaustive three-day course was considered to be an excellent ratio of days: topics, 100% felt this was an excellent ratio by delegates, excellent (55%) or very good (45%), with supplementary homework style multiple-choice questions provided at the end of each day. Given the multivariate aspects influencing success rate, a course such as this serves to maximise these chances and provide cohesive clinical revision as well as reduce the potential financial burden of resit exams.

The course is organised in such a way that delegates can pay for separate days if they are unable to attend all three or if they feel strongly about certain themed days, enabling a course tailored to the individual’s strength. It fulfils its aim of covering the MRCS Part A curriculum succinctly whilst communicating across key concepts for sitting the exam. The faculty members were welcoming, with 100% of delegates regarding the academic standard as well as the friendliness of faculty to be above average, and despite the COVID-19 lockdown, provided formal documentation enabling ease of travel for delegates. Coupled with extensive positive reception across three years’ feedback, these Basic Sciences and Clinical Application for MRCS A course swiftly demonstrates the benefits of attending to those with a career in surgery in mind, indeed, dissecting the first great barrier into higher training that is the MRCS.
